# Diffusion kurtosis imaging and pathological comparison of early hypoxic–ischemic brain damage in newborn piglets

**DOI:** 10.1038/s41598-020-74387-0

**Published:** 2020-10-14

**Authors:** Juan Xiao, Xiaoning He, Juan Tian, Honghai Chen, Jing Liu, Chao Yang

**Affiliations:** 1 Department of Radiology, The Second Affiliated Hospital of Dalian Medical University, No. 467 Zhongshan Road, Shahekou District, Dalian, Liaoning China; 2 Dalian Medical University, No. 9, West Section, South Lvshun Road, Dalian, Liaoning China

**Keywords:** Diseases, Medical research

## Abstract

To investigate the application value of magnetic resonance diffusion kurtosis imaging (DKI) in hypoxic–ischemic brain damage (HIBD) in newborn piglets and to compare imaging and pathological results. Of 36 piglets investigated, 18 were in the experimental group and 18 in the control group. The HIBD model was established in newborn piglets by ligating the bilateral common carotid arteries and placing them into hypoxic chamber. All piglets underwent conventional MRI and DKI scans at 3, 6, 9, 12, 16, and 24 h postoperatively. Mean kurtosis (MK) and mean diffusivity (MD) maps were constructed. Then, the lesions were examined using light and electron microscopy and compared with DKI images. The MD value of the lesion area gradually decreased and the MK value gradually increased in the experimental group with time. The lesion areas gradually expanded with time; MK lesions were smaller than MD lesions. Light microscopy revealed neuronal swelling in the MK- and MD-matched and mismatched regions. Electron microscopy demonstrated obvious mitochondrial swelling and autophagosomes in the MK- and MD-matched region but normal mitochondrial morphology or mild swelling in the mismatched region. DKI can accurately evaluate early ischemic–hypoxic brain injury in newborn piglets.

## Introduction

Neonatal hypoxic–ischemic encephalopathy is one of the major diseases causing neonatal death and permanent sequelae. Its global incidence among human newborns is approximately 1‰–3‰. This disease results in a serious burden on affected patients as well as their families^1–4^. Therefore, early and accurate assessment of the lesion is critical for predicting the patient’s prognosis.

Diffusion-weighted imaging (DWI) has been widely used for the early diagnosis of neonatal hypoxic–ischemic brain damage (HIBD). Although DWI and its associated parameter, i.e., apparent diffusion coefficient (ADC), can effectively detect early hypoxic injury, previous studies have shown that DWI and ADC often underestimate the extent of the lesions^5,6^. Diffusion-weighted imaging is a technique used for mono-exponential models. Water molecules are considered to be freely diffused in a Gaussian distribution^7^. However, the complex structures of biological tissues, such as cell membranes and organelles, affect the diffusion of water molecules, and their diffusion displacement and distribution deviate from the Gaussian distribution pattern. Diffusion kurtosis imaging (DKI), an advanced diffusion imaging technique, corrects the defects of the Gaussian model, quantifies the deviation of each voxel from free diffusion, and is sensitive to the heterogeneity of water molecule diffusion^8,9^. Research suggests that DKI more accurately reflects changes in tissue microstructure^10–12^, and parameters of both kurtosis and diffusion tensor imaging can be derived^8^. The most commonly used DKI-derived parameters are mean kurtosis (MK) and mean diffusivity (MD). MK is the mean value of diffusion kurtosis for each spatial gradient and is a dimensionless parameter that reflects the degree of diffusion limitation. MD reflects the mean value of the multidirectional diffusion of molecules corrected by non-Gaussian distribution^13,14^.

In the study of ischemic stroke^15–19^, MD is thought to reflect the extent of cytotoxic edema in tissues. The MK of DKI-derived parameters is sensitive to the cytoskeletal structure of cells that rupture and are deformed due to mitochondrial edema and increase in viscosity of the cell fluid. Some scholars have proposed that the combination of MK and MD can help accurately determine the infarct core and ischemic penumbra^15–17^. Our study aimed to investigate the application value of magnetic resonance diffusion kurtosis imaging (DKI) in the microstructural evaluation of HIBD in newborn piglets and to compare imaging with pathological results.

## Results

### MRI performance

#### The temporal evolution and percent changes of MK and MD

**Figure 1:**
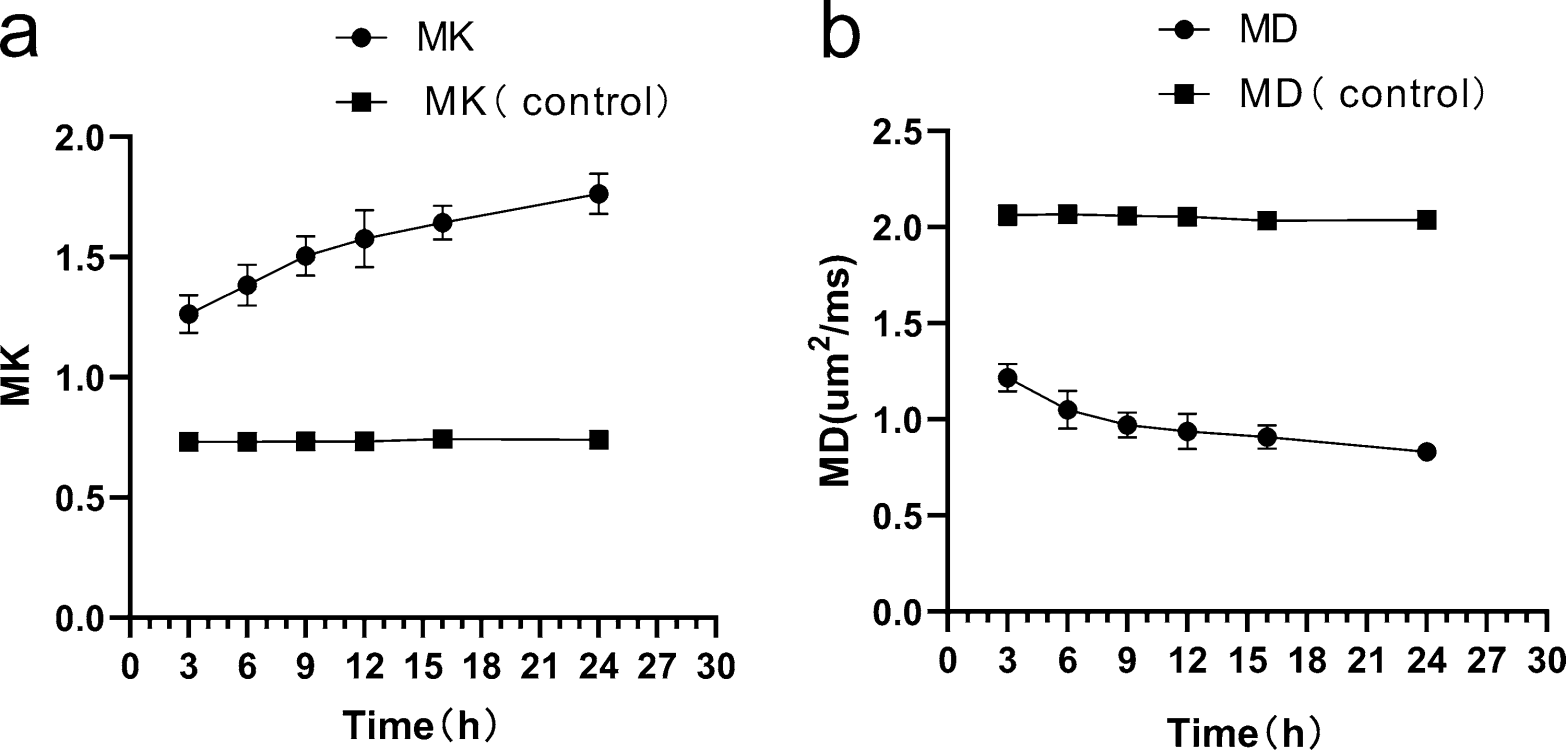
MK and MD values of the experimental and control groups over time. The abscissas represent the lengths of time after the induction of hypoxia; the ordinates represent MK and MD values.

**Figure 2:**
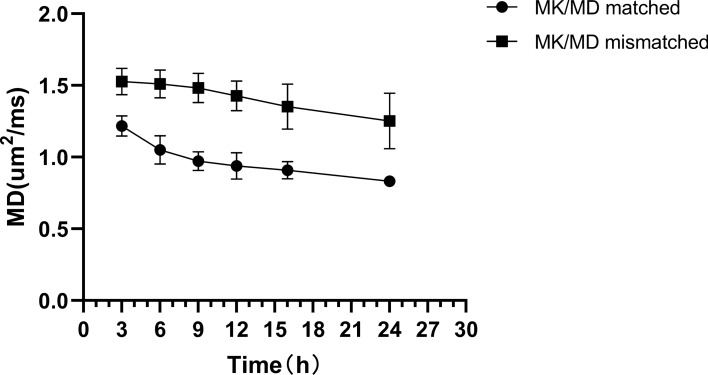
The MD values of the MK- and MD-matched and MK- and MD-mismatched regions over time.

The majority of the lesions in this study were located in the subcortical white matter and lateral paraventricular areas. Compared with that in the normal control group, the MD value in the lesion area gradually decreased from 3 to 24 h after ischemia and hypoxia, whereas the MK value gradually increased in the experimental group, with a statistically significant difference (*p* < 0.001). MK and MD values changed quickly in the 3–12 h period and slowly in the 12–24 h period (Fig. 1 and Table 1). The MD value in the MK- and MD-matched regions was significantly higher than that in the mismatched regions during each measurement timepoint (Fig. 2).

**Table 1:** Comparison of MK and MD values between experimental and control animals at different timepoints. MK and MD parameter values are expressed as means ± standard deviations. MK (control) represents the control group’s MK value; MD (control) represents the control group’s MD value.

Value	Time (h) after induction of hypoxia						*p*
	3	6	9	12	16	24	
MK	1.26 ± 0.08	1.38 ± 0.09	1.51 ± 0.08	1.58 ± 0.12	1.64 ± 0.07	1.76 ± 0.08	< 0.001
MK (control)	0.73 ± 0.01	0.73 ± 0.01	0.73 ± 0.02	0.73 ± 0.01	0.74 ± 0.01	0.74 ± 0.01	> 0.05
*t*	27.357	31.922	38.766	29.954	54.647	48.118	
p*	< 0.001	< 0.001	< 0.001	< 0.001	< 0.001	< 0.001	
MD	1.22 ± 0.07	1.05 ± 0.10	0.97 ± 0.07	0.93 ± 0.09	0.91 ± 0.06	0.83 ± 0.04	< 0.001
MD (control)	2.06 ± 0.05	2.07 ± 0.03	2.06 ± 0.05	2.05 ± 0.02	2.03 ± 0.02	2.04 ± 0.03	> 0.05
*t*	− 44.548	− 41.352	− 59.214	− 50.134	− 67.961	− 98.441	
p*	< 0.001	< 0.001	< 0.001	< 0.001	0.002	< 0.001	

*p*: Among different time points; p*: experimental group vs. control group.

**Figure 3:**
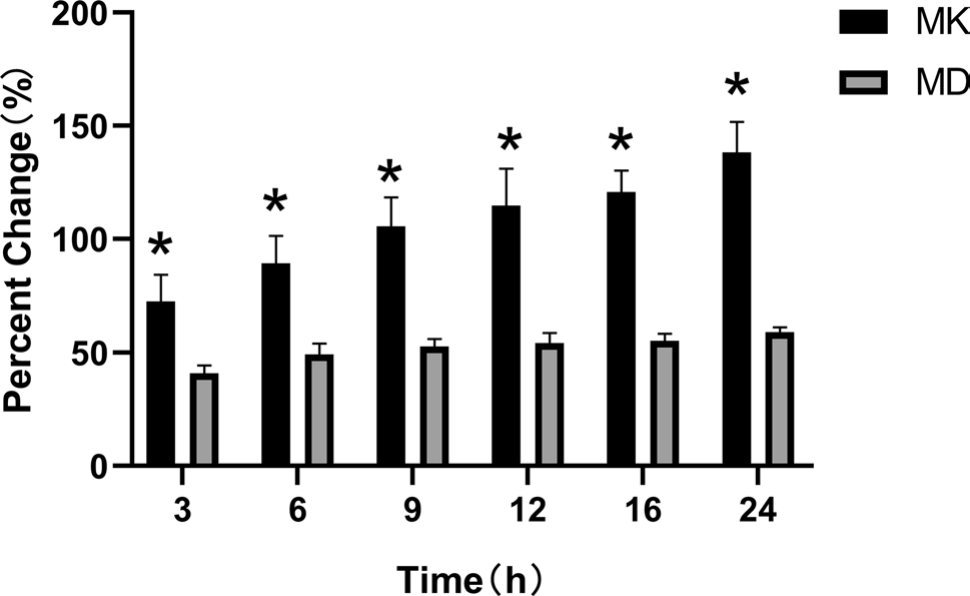
Change rate in percentages of MK and MD values at each time of measurement in the experimental group. The asterisk (*) indicates statistical significance.

In the experimental group, the percent change of MK was significantly higher than that of MD at different time points (Fig. 3).

#### The lesion areas on MK and MD

**Figure 4:**
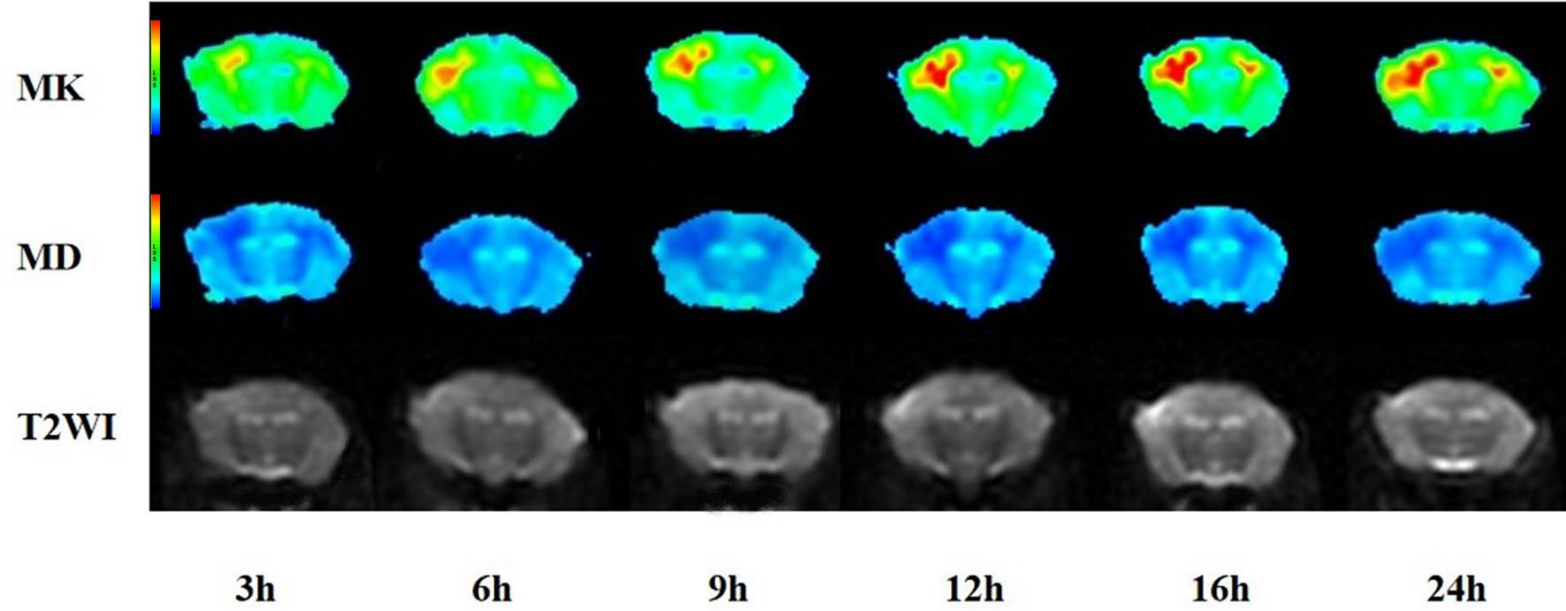
Representative maps of MK (the first row), MD (the second row), and T2WI (the third row) of the lesions, obtained from a single animal in the experimental group at different imaging time points (3, 6, 9, 12, 16, and 24 h postoperatively). Over time, MK and MD areas of the lesions gradually increased. The MD area at each imaging time was always greater than the MK area.

**Figure 5:**
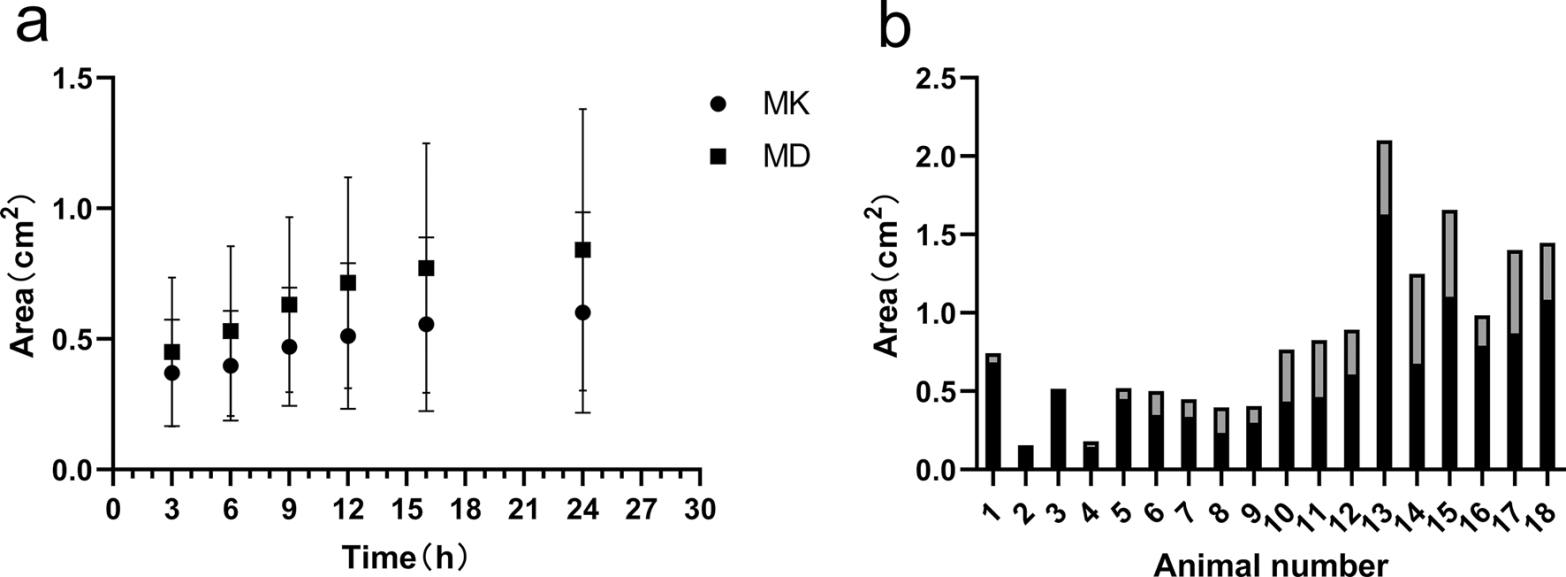
(**a**) Areas of MK and MD maps for the lesions in the experimental group over time. The abscissas represent the lengths of time after the induction of hypoxia; the ordinates represent the lesion areas on MK and MD maps (in square millimeters). Error bars represent the range of lesion area in 18 newborn piglets. **b.** Areas of MK and MD maps for the lesions in the experimental group at 24 h. Gray bar diagrams show that the MD area is greater than the MK area.

On MK and MD maps (Fig. 4), the lesion areas in the experimental group gradually increased with time (*p* < 0.001). The signal on the MK map was heterogeneous, whereas the signal on the MD map was relatively homogeneous. The range of regions with abnormal signals on MK maps of different individual animals at different measurement timepoints was smaller than that of regions with abnormal signals on MD maps (Fig. 5). This difference was also statistically significant (*p* < 0.005). Detailed results are listed in Table 2.

**Table 2:** Comparison of MK and MD lesion areas (cm^2^) at different timepoints in the experimental group. *MD* mean diffusivity, *MK* mean kurtosis.

Value	Time (h) after induction of hypoxia						*p*
	3	6	9	12	16	24	
MK	0.371 ± 0.204	0.399 ± 0.210	0.470 ± 0.227	0.512 ± 0.279	0.557 ± 0.332	0.602 ± 0.384	< 0.001
MD	0.450 ± 0.286	0.530 ± 0.325	0.631 ± 0.335	0.716 ± 0.404	0.772 ± 0.478	0.842 ± 0.539	< 0.001
*t*	− 3.787	− 4.441	− 5.623	− 5.476	− 4.725	− 5.141	
p*	0.002	< 0.001	< 0.001	< 0.001	< 0.001	< 0.001	

### Pathological examination

**Figure 6:**
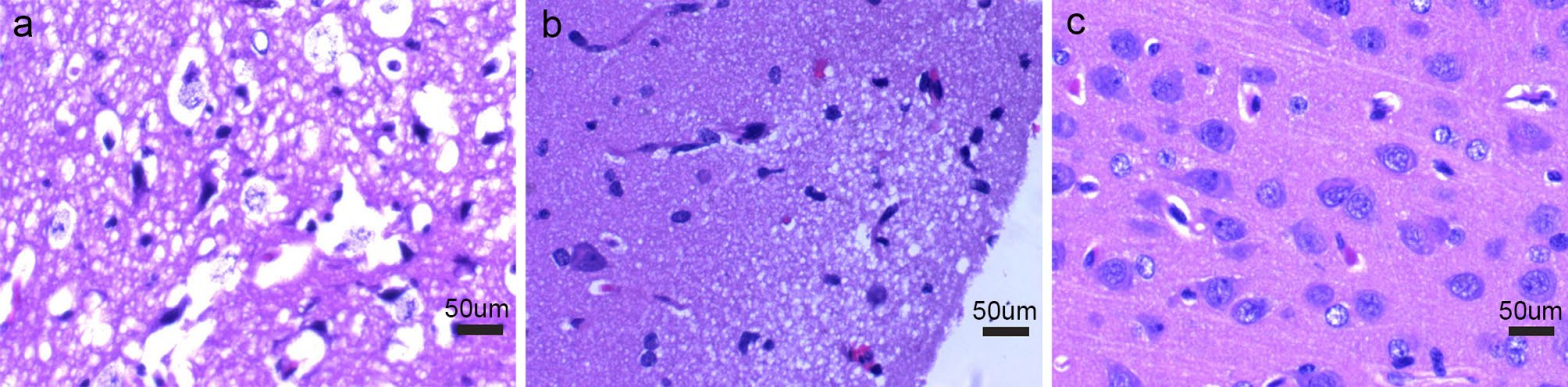
Pathological findings of lesions and normal tissues by light microscopy in piglets at the 24-h time point. **a.** Pathological microscopic phenomenon of MK- and MD-matched region in the right corona radiata in the experimental group 24 h postoperatively (× 600). All of the glial cells are swollen, some of the nuclei have disintegrated and are necrotic. **b.** Light microscopic appearance of MK- and MD-mismatched region (× 600). Glial cells are mildly swollen. **c**. Normal control group (× 600).

Compared with normal histopathology, pathological tissue alterations were observed in both MK and MD matching or mismatched regions. In both MK- and MD-matched regions or mismatched regions, light microscopy revealed the swelling of glial cells and neurons, a large number of inflammatory cell infiltrates. The extent of cell swelling in the MK- and MD-matched regions was greater than that in mismatched regions, characterized by karyopyknosis, eosinophilic cytoplasm, and intercellular narrowing (Fig. 6), as well as local necrotic changes at the center of the lesions in MK- and MD-matched regions.

**Figure 7:**
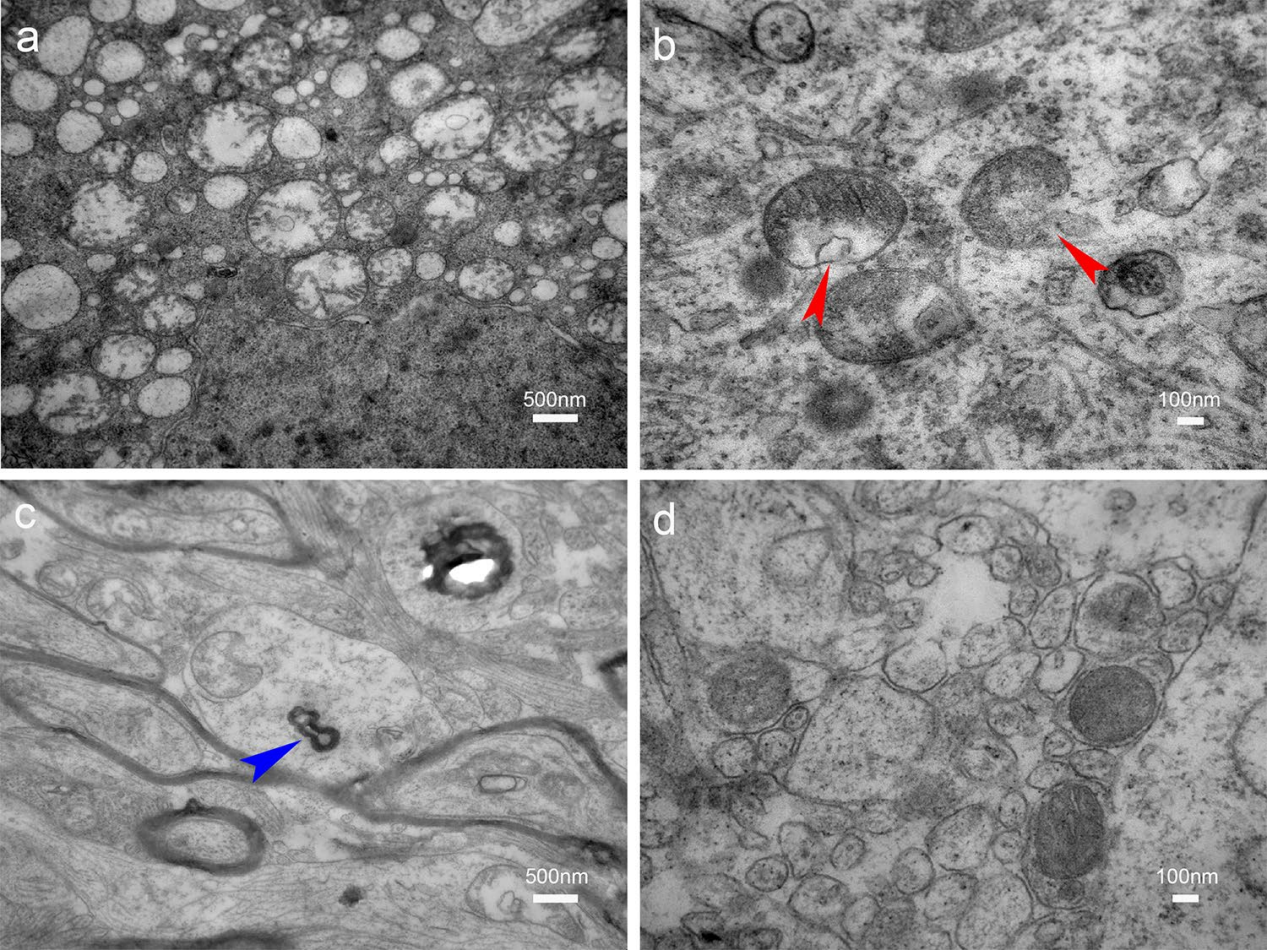
Pathological findings of lesions and normal by electron microscopy in a piglet at the 24-h time point. **a.** MK- and MD-matched region **(**× 25,000**),** the mitochondria with different degrees of swelling. **b.** MK- and MD-matched region **(**× 80,000**),** partial mitochondrial membrane collapse and rupture (red arrows). **c.** MK- and MD-matched regions **(**× 25,000**),** intracellular autophagosome changes (blue arrow). **d.** MK- and MD-mismatched region (× 80,000), organelles are acceptable, and some are only slightly swollen.

Electron microscopy was used to observe the MK- and MD-matched regions and mismatched regions (Fig. 7). The MK- and MD-matched region revealed different degrees of mitochondrial swelling, some mitochondrial membrane collapse and rupture, blurred and broken mitochondrial ridges, and autophagosomes (Fig. 7a–c). Conversely, in the MK- and MD-mismatched region, most mitochondrial structures were normal, with only a few exhibiting mild swelling (Fig. 7d).

## Discussion

HIBD decreases the cerebral blood flow, which results in anaerobic cell metabolism, followed by cell edema, energy failure, and various grades of metabolic encephalopathy^20,21^. Some studies confirmed that the lesions continue to expand during the early stages of HIBD^22,23^, a result consistent with the results of this experiment; thus, internal structural changes of such lesions should be accurately assessed. Due to the strong homology between pig and human brain tissues^24^, newborn piglets were selected as the subjects of this experiment, and the HIBD model was successfully established in piglets in the previous experiment, and corresponding results were obtained^22^.

Currently, kurtosis parameters are considered more sensitive than diffusion parameters for the evaluation of ischemic lesions by DKI^18,25^. MD is a comprehensive imaging measure of the dispersion of certain voxels or a region of a tissue. It reflects the overall level of molecular diffusion and diffusion resistance. Many studies have shown that MD is a reflection of the relative uniformity of the lesion signal but does not directly reflect the different metabolic conditions within the lesion^13,26^. In the final analysis, the diffusion coefficient can reflect only the pathological level of cerebral edema but not the damage of organelles and cell structures in cells. As the most specific parameter of DKI, MK is considered an indicator of the complexity of tissue microstructure, which can reflect the complexity and heterogeneity of the cell microenvironment^26^.

In this study, most of the lesions were located in the subcortical white matter and lateral paraventricular areas because the blood flow was redistributed to the brain area with high metabolism during ischemia and hypoxia. Our results showed that the MK value in the lesion area gradually increased and the MD value gradually decreased, which proved that HIBD gradually increased during the time between the 3 h and 24 h measurements. This finding is consistent with the results of Zhang et al.^17^, which suggest that the lesion continues to deteriorate. The degree of change in that period of 3–12 h is relatively large; during the period of 12–24 h, the change is relatively minimal. This also indicates that ischemic and hypoxic brain cytotoxic edema rapidly progresses from 3 to 12 h. Therapeutic hypothermia is no longer protective once seizures prominently occur at this time^27^, and thus, early clinical intervention should be performed.

Although the change during the period of 12–24 h is slow, the authors speculate that the degree of organelle damage during this stage is more serious and most of the organelles tend to deteriorate completely by the end stage. Moreover, the rate of change of the MK value in the lesion is significantly higher than that of the MD value, which indicates that the damage demonstrated by the MK value is more complex than that demonstrated by the MD value. Pathological results showed that the degree of organelle swelling and organelle structure destruction in the MK- and MD-matched region was more serious than that in the MK- and MD-mismatched region. The swelling and structural destruction of organelles make the water molecule diffusion more limited. The MK value is a more sensitive indicator of damage than the MD value, which suggests that the former can be used to represent the microenvironment complexity index and water molecule heterogeneity in the tissues^16,18,28,29^. In calculating the rate of change of MK and MD values, and to avoid bilateral infarction and the complication of side infarction and the influence of the contralateral perfusion pressure increase, we used measurements in a normal control group, which provided a more accurate scale for lesion evaluation.

In this study, the MK map showed that the lesions were uneven and produced high signals on imaging and that higher signals were often concentrated in the center of the lesion. The MD map showed relatively uniform signals, which is consistent with the findings of stroke studies^28,30^ and demonstrates that MK is a more sensitive indicator in lesion evaluation. This study also demonstrated that at each time of measurement of HIBD, the lesion area of the MD map was larger than that of the MK map, suggesting that MK is a more stable index in evaluating hypoxic–ischemic brain injury. In the study of stroke^15,16^, the MK and MD overlap region was defined as the infarct core, which is an irreparable region, and the MK and MD mismatch region was the ischemic penumbra, representing salvage tissue. Based on these characteristics, we performed a pathological examination; further, light microscopy analysis revealed that the MK- and MD-matched and MK- and MD-mismatched regions had basically the same characteristics, with cell edema as the main manifestation. These were the pathological bases for the MD abnormality. Remarkably, the degree of cell swelling in the MK- and MD-matched region was slightly higher than that in the mismatched region, which also explains that the MD value in the matched region was lower than that in the mismatched region.

However, electron microscopic study revealed different degrees of mitochondria swelling and mitochondrial membrane rupture and collapse in the MK- and MD-matched regions, which verified that the matched region was severely damaged and had seriously affected the damage of the organelle. A previous study found that autophagy flux increases in the early stage of ischemia and hypoxia, and later results in lysosomal dysfunction that damages the autophagy clearance^31^. Autophagosomes were also found in the MK- and MD-matched region, which further suggested that cell damage initiated autoapoptosis, confirming that the matched region was in an irreversible state. However, MK- and MD-mismatched regions showed normal or mild swelling of the mitochondria. This finding confirmed that the MK- and MD-mismatched region represents tissues with less damage that should concern the doctor and attempted to be rescued. Weber et al.^32^ and Lu et al.^33^ also confirmed that the mismatched regions can return to normal levels.

This study has certain limitations. The sample size was small; to ensure the status of newborn piglets, no scan was performed during the first 3 h after ischemia was induced; thus, the earliest appearance of damage was not observed; the scanning time was relatively short, and changes after 24 h and the MK and MD pseudo normalization time were not observed. These will be improved in subsequent experiments. Motion/eddy-current/susceptibility corrections, including possible outlier detection and correction, can lead to spurious findings. Multiple post-processing tools should be considered in future studies.

We evaluated the diagnostic value of DKI in evaluating HIBD in newborn piglets. We confirmed that MK and MD can give complementary diffusion information in HIBD. The DKI-derived parameters MK and MD can elucidate lesions stratification caused by ischemia and hypoxia from the organelle level. This may provide evidence for the DKI application to evaluate early neonatal hypoxic–ischemic encephalopathy.

## Methods

### Model establishment

All piglets were obtained in accordance with the guidelines of the National Institutes of Health, and the experimental animal protocol was approved by the Animal Care and Use Committee of Dalian Medical University. A total of 36 (18 male and 18 female) healthy newborn piglets (Dalian Huaqiao Breeding Swine Herd, Liaoning, China) aged 3–5 days and weighing approximately 2 ± 0.25 kg were randomly divided into experimental and control groups (*n* = 18 each). The room temperature was maintained at 28 °C ± 2 °C, and piglets were given milk powder feedings during the study period. Isoflurane inhalation was used to anesthetize the piglets intraoperatively. In the experimental group, the HIBD model was established. In each piglet, a longitudinal incision of approximately 2–3 cm was made in the median anterior cervical region, and the bilateral common carotid arteries were separated with permanent ligation using a 4.0 surgical line. Then, the piglet was placed into a tank where hypoxia was induced using a mixture of 4% oxygen and 96% nitrogen, administered at 2 L/min for 30 min. In the control group, only a sham operation was performed; those piglets were not subjected to bilateral common carotid artery ligation and hypoxia. Postoperatively, the animals were monitored in a recovery room, and a subcutaneous injection of morphine (0.3 mg/kg) was administered for pain relief. Gentamicin was also administered to prevent infection immediately after surgery.

### Magnetic resonance imaging

Magnetic resonance imaging (MRI) was performed using the Discovery MR750w 3.0T MR scanning device (GE Discovery MR750w) and a standard 32-channel head coil. Animals were positioned in a home-made wooden fixation box, and inhalation anesthesia was appropriately administered. Animals in both groups underwent conventional MRI (including T1- and T2-weighted imaging and T2-weighted fluid-attenuated inversion recovery imaging) and DKI scans at 3, 6, 9, 12, 16, and 24 h postoperatively. All sequences were scanned with coronal position. To ensure consistency of scanning layers at different time points of the same newborn piglet, the positioning line of each scan is maintained. DKI scanning involved a single-shot spin-echo imaging with the repetition time of 4500 ms and with minimal echo time; the direction of the diffusion-sensitive gradient was 20; the *b* values were 0, 1000, and 2000 s/mm^2^; the matrix was 128 × 128; and the field of view was 220 × 220 mm^2^. The layer thickness was 3.0 mm, the layer spacing was 0.5 mm, the number of excitations was 2, and the scanning time was 14 min and 45 s. Parameters for both DKI and diffusion tensor imaging were obtained simultaneously.

### Image postprocessing and analysis

After the DKI scan, the image data were transmitted to the Advantage Workstation 4.7 (GE Healthcare) and processed using the FuncTool software (GE Healthcare) to generate MK and MD maps. In the experimental group, the region of interest (ROI, area = 3 mm^2^) selected was the central area of the lesion, and the peripheral ambiguous area of the lesion was excluded as large as possible; in the control group, the ROI used for measurement was in the same location. Three ROIs were placed in each area, and measurements were averaged. The parameter characteristics of the lesions at different times were analyzed, and the change rate of each parameter value was calculated as percentage, as follows:

$${\text{Percentchange}}\;(\% ) \, = \, ({\text{ Parameter }}\;{\text{value }}\;{\text{of }}\;{\text{lesion}}\; - {\text{ Parameter }}\;{\text{ value }}\;{\text{of }}\;{\text{same }}\;{\text{area }}\;{\text{in }}\;{\text{control}}\;{\text{ group}})/{\text{ Parameter }}\;{\text{ value}}\;{\text{ of}}\;{\text{ same }}\;{\text{area}}\;{\text{ in }}\;{\text{control }}\;{\text{group }} \times \, 100\%$$

The image data were exported to the Digital Imaging and Communications in Medicine format, the lesion area of the MK and MD images at each time was measured using ImageJ software, and ranges of the two measurements were compared. The maximum layer of the lesion was selected for area measurement. Each lesion area was measured thrice and measurements were averaged. The MK- and MD-matched area was defined as the overlap area in which both the MD signal was anomalous and the MK signal was abnormal, whereas the MK- and MD-mismatched area was defined as the area in which the anomalous MD signal was significantly larger than the area of the abnormal MK signal. MD values of the MK- and MD-matched region and MK- and MD-mismatched region were measured, respectively.

All ROIs and lesion areas were measured independently by two neuroradiologists with 12 and 15 years of experience. They agreed on the outline of the image before measuring it, and then took the average of the two as the final data. The results were examined by a third neuroradiologist with 15 years of experience.

### Pathological examination

Animals from both groups were positioned prone on the operating table after the MRI examination. The brain tissues of 18 newborn piglets were randomly selected for pathological examination under light microscope in both experimental (n = 9) and control groups (n = 9). The brain tissues of the other 18 newborn piglets were observed under transmission electron microscope. Under inhalation anesthesia, the skull was opened to expose the brain tissue, and the brain tissue was completely removed after the brain stem was severed from the skull base. With regard to the scan line (the vertical line at the skull base), the location of the largest lesion in brain tissue was identified. The location of the largest lesion was cut into 3 mm thick slices with bacteria-free blade along the parallel optic chiasma direction and then fixed in 4% paraformaldehyde solution for 48 h. Based on MK and MD images, the lesion layer was measured from the edge of brain tissue with a ruler, and the MK- and MD-matched and mismatched regions were also measured, respectively. After the routine dehydration and transparent paraffin embedding, a pathological section with a layer thickness of approximately 4 μm was cut with a microtome; HE staining was performed; and then the brain tissues were observed under a light microscope.

After isolating the brain tissue from the body, the matched and mismatched regions were rapidly located according to the above methods. From the MK and MD matching and mismatched regions, tissue with a volume of approximately 1 mm × 1 mm × 1 mm was cut and removed using a sterilized toothpick and then fixed in a 2.5% glutaraldehyde solution at 4 °C in a refrigerator. To maintain cytoactivity, the slices should be rapidly kept in a 2.5% glutaraldehyde solution. After rinsing, dehydration, infiltration, and embedding polymerization, the tissue was cut into about 0.1 μm slices using an ultramicrotome and then stained with uranyl acetate and citric acid. These volumes were then observed with electron microscopy and digitally photographed.

The sampling locations of MK and MD matched and mismatched regions are shown in Supplementary Figure S1 (online).

### Statistical analysis

SPSS version 19.0 (IBM, Armonk, NY) was used to perform statistical analysis. Data were normally distributed and expressed as means ± standard deviations for each group. Differences of MK and MD values among different timepoints from 3 to 24 h were calculated using repeated-measures analysis of variance and post hoc least significant difference method tests. MK and MD values of the experimental and control groups, MD values of the matched and mismatched regions, and MK and MD regions of the experimental group at different timepoints were compared using paired-sample *t* test. *P* < 0.05 was considered statistically significant.

## Supplementary information

Supplementary Figure S1.
