# Supplementary material for: Diffusion kurtosis imaging and pathological comparison of early hypoxic–ischemic brain damage in newborn piglets
Source: Sci Rep. 2020 Oct 14;10:17242. doi: 10.1038/s41598-020-74387-0 (PMC7560608; doi:10.1038/s41598-020-74387-0)
Supplement: Supplementary file 1 — Supplementary Figure S1. [file 41598_2020_74387_MOESM1_ESM.pdf]

Diffusion Kurtosis Imaging and Pathological Comparison of Early Hypoxic–Ischemic Brain Damage in Newborn Piglets

Juan Xiao[1], Xiaoning He[1], Juan Tian[1], Honghai Chen[1], Jing Liu[2], Chao Yang[1*]

[1] Department of Radiology， The Second Affiliated Hospital of Dalian Medical University， China (No. 467 zhongshan road, Shahekou District, dalian, liaoning Province, China)

[2] Dalian Medical University， China (No.9,west section,South Lvshun Road,Dalian,Liaoning Province,China)

[*] Corresponding Author: Chao Yang; E-mail: dryangchao@163.com

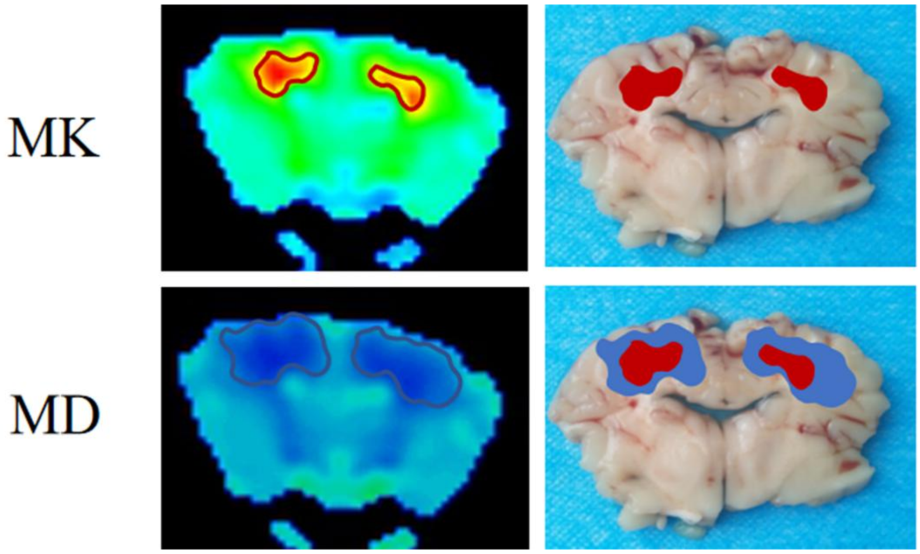

Supplemental Figure S1：

Pathological location: The red area represent MK- and MD-matched region and blue area represent mismatched region in brain tissue sections.
